# A Personal Tribute to the Late Dr. L. P. Haskell

**Published:** 1916-11

**Authors:** C. N. Johnson


### A PERSONAL TRIBUTE TO THE LATE DR. L. P. HASKELL.

BY C. N. JOHNSON, D.D.S.

I had known Dr. Haskell for thirty-two years, and in some respects he was the most remarkable man I have ever met. His was the common lot of most men, to be buffeted by fate at times, and yet, I never knew him to be anything but cheerful, hopeful and complacent. What other men took as a reverse he accepted as merely an incident in the day's work. Out of his inner consciousness there never emanated the miasma of doubt, or distrust or envy. He did not know how to hate. He attacked wrongs at times but never men, and in the alembic of his mind there never brewed the dregs of rancor.

He was one of the greatest optimists in the dental profession, and never in the darkest days of his ninety years of life was the light ever obscured from his point of view. As the span of man's age is measured, he had been old for years, but in his mentality he was ever young, and looked more toward the future than to the past.

He was favored physically, having come to his old age with few ills. His characteristic remark, when asked by a friend how he felt, was always: "The old craft is still afloat—timbers all sound—and no prospect of going into dry dock for repairs very soon." He never knew what it was to have a "cold," and when I talked with him at the hospital a short time before his death he assured me that it was the first time he had experienced an ache or pain in twenty-five years. Truly the machinery was tuned to run well and smoothly.

Maybe after all the mentality has much to do with the physical balance, because this man's mind was normal, serene and healthful always. The only complaint I ever heard him make—and this was more a lament than a complaint—was in his later days when his failing sight had incapacitated him for work. To be deprived of work was the greatest hardship he ever suffered. He said to me frequently: "To be obliged to sit around and do nothing is the hardest task I ever performed." But even this was said with a smile.

It had been his prediction to me many times that he would probably go to his office regularly and attend to his usual duties till suddenly the machinery would run down and he would never go again. He hoped to die in harness, and while this hope was hardly realized, yet he was mentally alert to the last, and died like a good soldier with his face toward the front.

His immediate friends will miss his pleasant personality, but his influence for good in his profession will last longer than personality, because of what he has said in the lecture room and what he has recorded on the printed page. His memory will ever remain a sweet savor with those who knew him well, and this in the ultimate is the most and best that can be said of any man. Hats off! A requiem for the Old Roman!—*Dental Review.*

